# Cryptic genetic variation and adaptation to waterlogging in Caledonian Scots pine, *Pinus sylvestris* L.

**DOI:** 10.1002/ece3.4389

**Published:** 2018-08-02

**Authors:** Kevin Donnelly, Stephen Cavers, Joan E. Cottrell, Richard A. Ennos

**Affiliations:** ^1^ Institute of Evolutionary Biology School of Biological Sciences Ashworth Laboratories University of Edinburgh Edinburgh UK; ^2^ NERC Centre for Ecology and Hydrology, Edinburgh Penicuik, Midlothian UK; ^3^ Forest Research Northern Research Station Roslin, Midlothian UK

**Keywords:** chlorophyll fluorescence, common garden, cryptic genetic variation, local adaptation, Scots pine, waterlogging

## Abstract

Local adaptation occurs as the result of differential selection among populations. Observations made under common environmental conditions may reveal phenotypic differences between populations with an underlying genetic basis; however, exposure to a contrasting novel environment can trigger release of otherwise unobservable (cryptic) genetic variation. We conducted a waterlogging experiment on a common garden trial of Scots pine, *Pinus sylvestris* (L.), saplings originating from across a steep rainfall gradient in Scotland. A flood treatment was maintained for approximately 1 year; physiological responses were gauged periodically in terms of photochemical capacity as measured via chlorophyll fluorescence. During the treatment, flooded individuals experienced a reduction in photochemical capacity, *F*_v_ */F*_m_, this reduction being greater for material originating from drier, eastern sites. Phenotypic variance was increased under flooding, and this increase was notably smaller in saplings originating from western sites where precipitation is substantially greater and waterlogging is more common. We conclude that local adaptation has occurred with respect to waterlogging tolerance and that, under the flooding treatment, the greater increase in variability observed in populations originating from drier sites is likely to reflect a relative absence of past selection. In view of a changing climate, we note that comparatively maladapted populations may possess considerable adaptive potential, due to cryptic genetic variation, that should not be overlooked.

## 1 INTRODUCTION

One of the key factors affecting the survival probability of plant populations in the face of global climate change will be their ability to adapt genetically to the novel abiotic and biotic conditions that they will encounter in the future (Gonzalez, Ronce, Ferriere, & Hochberg, 2013; Hoffmann & Sgrò, 2011). This ability will largely be governed by the genetic variance in fitness that they exhibit in the new environments into which they transition.

Genetic variance for fitness within a population is due to the presence of variation at many loci throughout the genome, each with the potential to alter the phenotype. A proportion of genetic variants in a population will have phenotypic effects expressed under present environmental conditions. If their effects on fitness are sufficiently large in relation to the effective population size, they will be exposed to natural selection (Eyre‐Walker & Keightley, 2007; Kimura, 1968; Rockman, 2012). Alleles that code for phenotypes mismatched to the environmental conditions are likely to decline in frequency, leading to a reduction in genetic variation and in the overall variance in fitness of the population.

However, in addition to such allelic differences expressed in current environments, there will be other genetic variants that have no phenotypic effects under current conditions, but will significantly influence the phenotype of individuals growing in novel environments (Gibson & Dworkin, 2004; Paaby & Rockman, 2014). These “cryptic” genetic variants are selectively neutral in the current environment, and their abundance in the population is governed solely by a mutation–drift balance (Hermisson & Wagner, 2004). Their presence in the population constitutes a store of *cryptic genetic variation* (Gibson & Dworkin, 2004; Le Rouzic & Carlborg, 2007; Paaby & Rockman, 2014). We note that although this term can also be used to describe variation exposed by “gene‐by‐gene” (GxG) interactions, whereby the penetrance of an allele may vary across different genetic backgrounds (Paaby & Gibson, 2016); in this paper, we are concerned solely with the release of variation via environmental novelty (GxE interaction).

Transfer of the population to a novel environment will cause some of the previously cryptic genetic variants to influence the phenotype (Schlichting, 2008), generating more genetically based phenotypic variation on which selection can act. This will enhance the potential rate of evolution (Kopp & Matuszewski, 2014; Ledon‐Rettig, Pfennig, Chunco, & Dworkin, 2014; McGuigan & Sgrò, 2009). Thus, cryptic variants form the portion of existing standing genetic variation, which ceases to be selectively neutral upon transition to a new environment. The presence of existing cryptic genetic variation in the population facilitates evolution in the new environment because adaptation is not solely reliant upon the emergence of beneficial new mutations, which arise uniquely within populations and are more likely to be lost than to be picked up by selection (Paaby & Gibson, 2016).

The presence and importance of cryptic genetic variation for response to selection under environmental change was recognized long ago by Mather (1942). Preliminary evidence for its existence in plant populations has come from the detection of increases in within‐population phenotypic variation when populations are experimentally transplanted into novel environments (Clausen, Keck, & Hiesey, 1940; Helgadottir & Snaydon, 1986; Schlichting, 2008). In addition, Cooper (1954) showed that artificial selection on cryptic genetic variation for flowering time, exposed by experimentally imposed environmental changes, can lead to rapid genetic divergence of populations.

The presence of cryptic genetic variation in natural populations and its role in facilitating adaptation to rapid environmental change have been widely recognized. However, the extent to which cryptic genetic variation may deliver enhanced adaptation to contemporary climate change remains unclear for most species. To assess this, we require detailed empirical measurements of the fitness responses that accompany transfer of populations into the range of novel environments anticipated under realistic climate change scenarios (Ledon‐Rettig et al., 2014). In this study, we report the results of an experiment that analyzes the fitness response of Caledonian Scots pine (*Pinus sylvestris* L.) populations to growth in waterlogged soil, an environment whose frequency is predicted to increase in Scotland under climate change (Murphy et al., 2009).

The Caledonian pine populations of Scotland comprise some 82 highly fragmented stands of *P. sylvestris,* which nonetheless retain high levels of both neutral and adaptive genetic variation, and a substantial degree of genetic connectivity (Kinloch, Westfall, & Forrest, 1986; Salmela, Cavers, Cottrell, Iason, & Ennos, 2011; Steven & Carlisle, 1959; Wachowiak, Iason, & Cavers, 2013; Wachowiak, Salmela, Ennos, Iason, & Cavers, 2011). Populations are distributed across a steep, longitudinal rainfall gradient, ranging from 3,000 mm/year in the west to 800 mm/year in the east, those in the west inhabiting a milder climate at lower average elevations. Furthermore, differences in underlying geology generate soils that are generally more freely draining in the east than in the west (Mason, Hampson, & Edwards, 2004). Previous work has demonstrated genetic differences among these populations for cold tolerance, phenology, needle characters, and pathogen tolerance that are consistent with adaptation to the environmental variation experienced across Scotland (Donnelly, Cavers, Cottrell, & Ennos, 2016; Perry et al., 2016; Salmela, Cavers, Cottrell, Iason, & Ennos, 2013; Salmela et al., 2011). Here, we investigate whether there is evidence for local adaptation for tolerance of waterlogging among present‐day Caledonian pine populations, and assess the extent of cryptic genetic variation for the trait under flooded conditions.

We exposed saplings, grown from seed collected in populations across the natural longitudinal range of Caledonian pine, to contrasting nonflooded and flooded treatments in a controlled experiment. We then measured a proxy for the fitness of individuals under these two treatments and compared the mean fitness of populations, and the variation in fitness among individuals within these populations. As, for practical reasons, it was only possible to use relatively young plants in this study, and for a fraction of their life cycle, a rapid and nondestructive method was required for measuring relative fitness that can be applied to a large sample of plants.

For this purpose, we measured the dark‐adapted chlorophyll fluorescence ratio (*F*_v_ */F*_m_) that estimates the relative efficiency of chlorophyll photosystem II (PSII) (Genty, Briantais, & Baker, 1989; Kautsky & Hirsch, 1931). The rationale is that if plants are of low fitness under a particular set of environmental conditions, this photosystem will be impaired, leading to a reduction in the *F*_v_ */F*_m_ ratio (Maxwell & Johnson, 2000). The approach has proven very effective in documenting the fitness response of a variety of plant species to novel environmental conditions including those of high and low temperatures (Gamon & Pearcy, 1989; Groom & Baker, 1992), freezing injury (Lindgren & Hällgren, 1993), and chemical pollutants (Strand, 1993). Water stress has also been investigated by means of chlorophyll fluorescence, chiefly in terms of drought (Demmig, Winter, Krüger, & Czygan, 1988; Garg et al., 2002; Huang & Gao, 1999), but also waterlogging. Pearson et al. (2013) conducted a three‐month combined drought–waterlogging experiment on Scots pine seedlings, concluding that both treatments adversely affected *F*_v_ */F*_m_, but that within the time frame of the experiment, the effects of drought wore more severe and, unlike waterlogging, also impacted upon growth traits. Repo et al. (2016) have also provided good evidence of a strong relationship between *F*_v_ */F*_m_ and maximum photosynthetic assimilation rate of Scots pine when exposed to waterlogged conditions.

Using this experimental protocol, we can ask a number of questions. The first is whether there is an overall effect of the flood treatment on our proxy of fitness. Our expectation is that across all populations there will be a significant reduction in the *F*_v_ */F*_m_ ratio under waterlogging. The second question is whether the mean reduction in fitness is affected by the origin of the sample. Our prediction is that the reduction in *F*_v_ */F*_m_ ratio will be lower in plants from populations that have experienced higher rainfall, where exposure to waterlogging is historically likely to have been greater. The third question is whether there is any evidence that cryptic variation in fitness is expressed under the novel environmental conditions. This would be seen as greater variation in the *F*_v_ */F*_m_ ratio in populations under the flood treatment. Lastly, we can ask whether such cryptic variation has been important in contributing to adaptation to waterlogging. If this were true, we would expect that under the flood treatment, variation in the *F*_v_ */F*_m_ ratio will be lower in plants from western/high rainfall populations, which we anticipate have been subjected to greater selective pressure from waterlogging than those from eastern/low rainfall populations.

## 2 METHODS

### 2.1 Plant material

**Figure 1:**
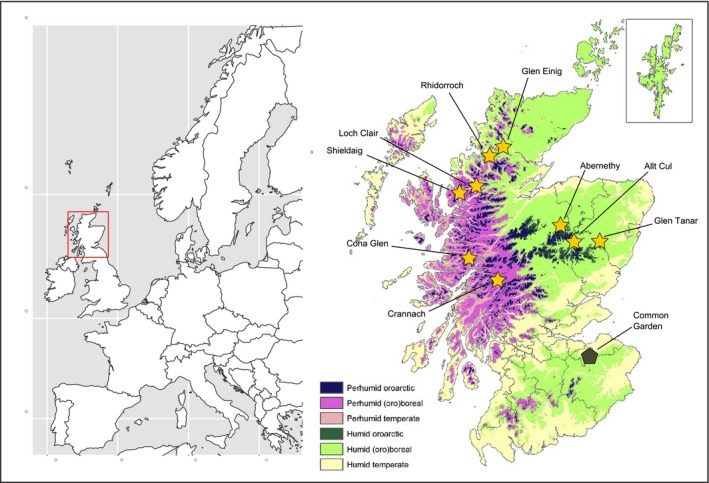
Sites of origin for the populations in the study and the location of the common garden. Map of bioclimatic zones adapted from Brown (2017), based upon data from 1991 to 2010 [Colour figure can be viewed at http://wileyonlinelibrary.com]

Seed was collected from nine native Scottish populations of *P. sylvestris* located across a steep rainfall gradient (Figure 1, Table 1) in March 2007. At each site, at least 20 cones were collected from each of four open‐pollinated trees located a minimum of 100 m apart. All materials were germinated and grown within the glasshouse. Seeds were sown in trays containing a mixture of 3:1 John Innes #1 compost:sand. Upon emergence of the first needle whorls in June 2007, seedlings were transferred to 0.62‐L pots. Following bud flush in 2008, seedlings were transferred to 1.5‐L pots containing John Innes #3. Each of the nine study populations was represented by four putative half‐sib families, with 12 individuals per family, yielding 432 individuals in total.

**Table 1:** Coordinates of trial populations, the range of altitudes at which the mother trees were sampled, alongside mean monthly temperature and rainfall taken from Met Office estimates

Provenance	Latitude	Longitude	Altitudinal range (m.a.s.l.)	Mean monthly temp (°C)	Mean monthly rainfall (mm)
Abernethy (AB)	57.21	−3.61	365–363	6.82	67.66
Allt Cul (AC)	57.04	−3.35	435–512	6.44	79.85
Cona Glen (CG)	56.79	−5.33	89–180	8.45	181.73
Crannach (CR)	56.58	−4.68	258–338	6.10	211.07
Glen Einig (GE)	57.95	−4.76	45–69	7.04	111.18
Glen Tanar (GT)	57.05	−2.86	293–422	7.40	70.91
Loch Clair (LC)	57.56	−5.36	102–166	8.07	234.94
Rhiddoroch (RD)	57.89	−4.98	138–220	8.45	139.39
Shieldaig	57.51	−5.64	44–132	7.60	198.71
*Common Garden*	55.86	−3.21	**190**	7.08	77.73

### 2.2 Experimental design

Saplings were 4 years old at the time treatment commenced. Individuals were placed into 12 plastic basins (hereafter “plots”), such that each held 36 individuals (one member from each family, randomly ordered within plots). Half of the plots were assigned to the flood treatment, and the remainder to the control; the placement of treatment and control plots was alternated throughout the glasshouse across four benches in a chequerboard fashion.

Small overflow holes were made at intervals around the perimeter of all plots: In the control plots, these holes were made ~1 cm from the base of the containers to permit a shallow maximum water depth, while in the flooded plots, perforations were made higher to maintain a water depth of ~2 cm below soil surface (~10 cm depth). As a constant water depth was maintained in the treatment plots, the drainage properties of the soil were effectively negated. The flooding regime began on July 5, 2011, with water levels maintained by an automated system.

### 2.3 Measurement of chlorophyll fluorescence

Measurements of dark‐adapted chlorophyll fluorescence were based on methodology developed by Kautsky & Hirsch (1931). A brief period of darkness allows PSII reaction centers to reset to an “open” state, that is, in a position to absorb actinic light. A short, high‐intensity pulse of light is then given with the effect of closing all available PSII reaction centers; it is during this period that the maximum fluorescence is emitted, termed *F*_m_. In conjunction with a measurement of the minimum fluorescence yield in the absence of actinic light, *F*_o_, the “variable fluorescence,” or *F*_v_, is calculated as *F*_v_ **= *F*_m_ **− *F*_o_. The ratio *F*_v_ */F*_m_ provides a sensitive measure of the maximum efficiency of PSII, shown to be ~0.8 across a wide range of plants (Björkman & Demmig, 1987).

*F*_v_ */F*_m_ was measured for all 432 individuals on 16 occasions throughout the trial using a *Hansatech Instruments Handy Pea* chlorophyll fluorimeter. The first measurements were on July 7, 2011, 3 days after the initiation of the flood treatment, and the last on October 8, 2012. Measurements were typically obtained between 13:00 and 17:00, requiring on average 3 hrs to complete. Individual needles were detached at random from the main stem of each plant and dark‐adapted for a period of at least 10 min before fluorescence measurements were taken. It has been shown that removal of needles prior to measurement has a negligible effect on the result (Mohammed, Binder, & Gillies, 1995). Previous‐year needles were initially used, as current‐year needles had not yet matured. After the sixth set of measurements made on September 15, 2011, all readings were taken from needles, which had emerged in spring 2011. Measurements on previous‐year needles were recorded biweekly; following the transition to current‐year needles, data were collected on a monthly basis.

### 2.4 Statistical analysis

Linear mixed‐effects models were employed during analysis: To achieve consistency with model assumptions, the *F*_v_ */F*_m_ ratio was odds‐transformed to improve the normality of the residuals:

(1) $$\text{Odds‐transformed}\;F_{v}/F_{m}=\frac{F_{v}/F_{m}}{1-(F_{v}/F_{m})}$$

All analyses were performed in *R* (http://www.R-project.org/). Mixed‐effects models were fitted using the package “lme4” (Bates, Maechler, Bolker, & Walker, 2015) by maximum likelihood (ML), and model subsets containing all possible combinations of fixed effects (with exception, see below) were generated using “MuMIn” (Barton, 2013). The extent to which fixed effects and their interactions improved model fit was evaluated in terms of the corrected *Akaike information criterion*, AIC_c_. This systematic evaluation of fixed‐effects combinations is similar to that described by Burnham and Anderson (2002) and Grueber, Nakagawa, Laws, and Jamieson (2011). Rainfall and longitude at site of origin were included as covariates, the latter having previously shown potential to be a more effective predictor of trait variation in Scotland than raw meteorological data (Donnelly et al., 2016). The best models were then refitted using restricted maximum likelihood.

### 2.5 Effects of flood treatment and sample origin on *F*_v_ */F*_m_

A longitudinal model was fitted as follows:

(2) Y=μ+T+C+D+T:D+T:C+C:D+d+p+f+i+pl+ε

where treatment group (*T*), a covariate (*C*) being one of mean monthly rainfall at site of origin in mm or longitude in degrees, days since treatment start (*D*), and their interactions were fixed effects; population (*p*), family within population (*f*), individual within family (*i*), and plot within treatment (pl) were random effects. Days since treatment start (*D*) was fitted both as a covariate (fixed effect) and as a random effect (*d*) and, being on a very different scale to other variables, was standardized (divided by two standard deviations) prior to analysis. As strong negative correlation exists between rainfall and longitude in Scotland, models which included both terms were excluded from consideration.

### 2.6 Effects of flood treatment and sample origin on variation in *F*_v_ */F*_m_ within populations

To determine whether variation in *F*_v_ */F*_m_ differed between treatments, the coefficient of phenotypic variation, CV_P_, was first estimated. Variances were partitioned for each population within treatment groups at all time points via the following model:

(3) Y=μ+f+pl+ε

where family (*f*) and plot (pl) were random effects. Phenotypic variance (*V*_P_) was then estimated as the total variance excluding that attributable to plot. For all populations, CV_P_ was estimated for each population, treatment, and time point combination as follows:

(4) $${\text{CV}}_{P}=\frac{\sqrt{V_{P}}}{\mu_{\mathrm{trait}}}\times 100$$

where μ_trait_ is the mean trait value. Effects of flood treatment and sample origin on CV_P_ were evaluated as follows:

(5) Y=μ+T+C+D+T:D+T:C+C:D+d+p+ε

following the same nomenclature as Equation (2) (as CV_P_ is estimated at population level, family, individual, and plot terms were omitted).

### 2.7 Autocorrelation structures

When modeling time‐series data, the occurrence of temporal autocorrelation may allow for spurious results if unaccounted for. It is therefore appropriate to test whether an autoregressive error structure may improve model fit (Pollitt, Reece, Mideo, Nussey, & Colegrave, 2012). As these structures were not supported by lme4, models describing time‐series data were respecified using the package “nlme” (Pinheiro, Bates, Debroy, & Sarkar (2018). No trends were observed in residual autocorrelation, and the inclusion of corARMA structures did not improve the AICc of model fits. The results presented are therefore derived from the lme4 models described above, which lack autocorrelation terms.

## 3 RESULTS

### 3.1 Evaluating the importance of model parameters

Support for a given parameter is described in terms of ΔAIC_c_, defined here as the difference in AIC_c_ between the best model and the equivalent model lacking that parameter (note that models omitting a main effect necessarily exclude the corresponding interaction terms). A summary of the top models is given in Table 2, and comprehensive model comparison tables are provided in Table S1 in addition to complete model output for the best models in Table S2.

**Table 2:** Top models in terms of AICc for differing combinations of fixed effects. For brevity, only models with a ΔAICc < 2 are shown (comprehensive results are provided in Table S1)

Intercept	Tre	Long	Rain	Day	Tre:Long	Tre:Rain	Tre:Day	Long:Day	Rain:Day	*df*	ΔAIC_c_	*w*
(a) Odds‐transformed *F*_v_ */F*_m_ for previous‐year needles												
3.470	+	−	−	−0.132	−	−	+	−	−	10	0.00	0.391
(b) Odds‐transformed *F*_v_ */F*_m_ for current‐year needles												
3.721	+	−0.026	−	0.041	+	−	+	0.047	−	13	0.00	0.326
3.995	+	0.035	−	−0.170	+	−	+	−	−	12	1.26	0.174
(c) CV_P_ for previous‐year needles												
35.49	+	−	−	6.346	−	−	+	−	−	7	0.00	0.346
37.35	+	−	−0.013	6.346	−	+	+	−	−	9	1.97	0.129
(d) CV_P_ for current‐year needles												
24.68	+	−	6.676e−4	1.764	−	+	+	−	−	9	0.00	0.493
24.68	+	−	6.676e−4	0.156	−	+	+	−	0.011	10	1.80	0.200

*Notes*.

For any given model, the terms present are represented by either “+” (for factors) or the value of the coefficient (for covariates); the absence of a parameter is denoted “−.” Note that “days since treatment start” was standardized (divided by two standard deviations) prior to analyses. Longitude and rainfall terms were not permitted to occur within the same model. Tables a and b describe fixed effects for the odds‐transformed *F*_v_ */F*_m_; c and d for CV_P_ derived from the same data (estimated at the population level).

Key: Tre, treatment; Long, longitude (°); Rain, mean monthly rainfall at site of origin (mm); Day, days since treatment start; *df*, degrees of freedom; ΔAIC_c_, difference in AIC_c_ between current and best model; *w,* Akaike weight.

### 3.2 Effects of flood treatment and sample origin on *F*_v_ */F*_m_

**Figure 2:**
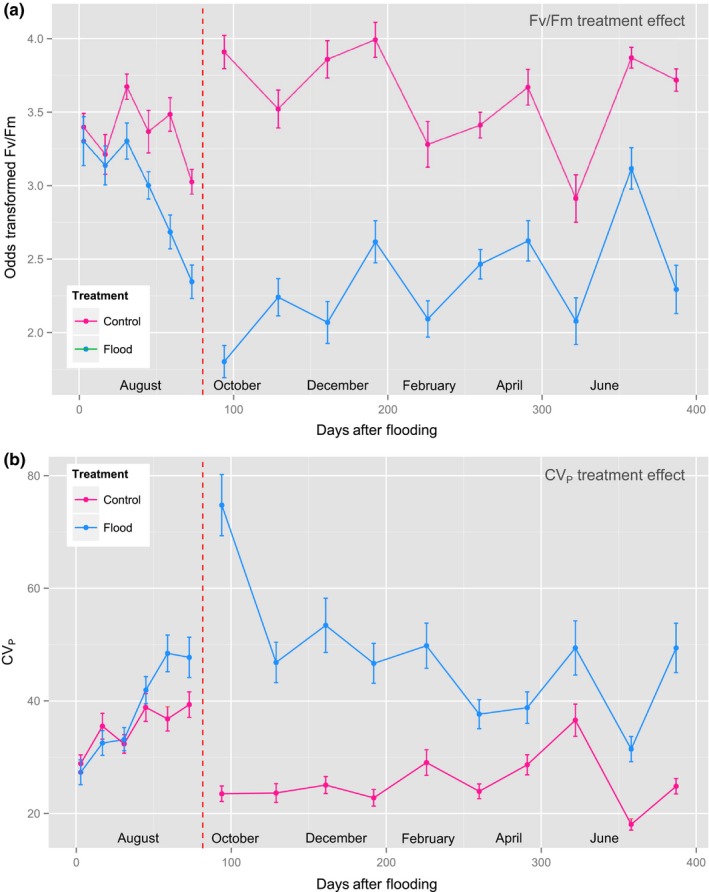
*F*_v_ */F*_m_ (odds‐transformed) for control and treatment groups throughout duration of study, for (a) mean values and (b) CV_P_. The transition between previous‐ and current‐year needles is represented by a line break. Photochemical capacity was reduced on average by exposure to waterlogging, whereas the reverse was true for CV_P_ [Colour figure can be viewed at http://wileyonlinelibrary.com]

In the initial period following flooding, during which measurements were made on previous‐year needles, *F*_v_ */F*_m_ values in the treatment group fell appreciably relative to the control (Figure 2a). There was very strong evidence for a treatment effect (ΔAIC_c_ = 59.92), but not for covariation with rainfall or longitude.

**Figure 3:**
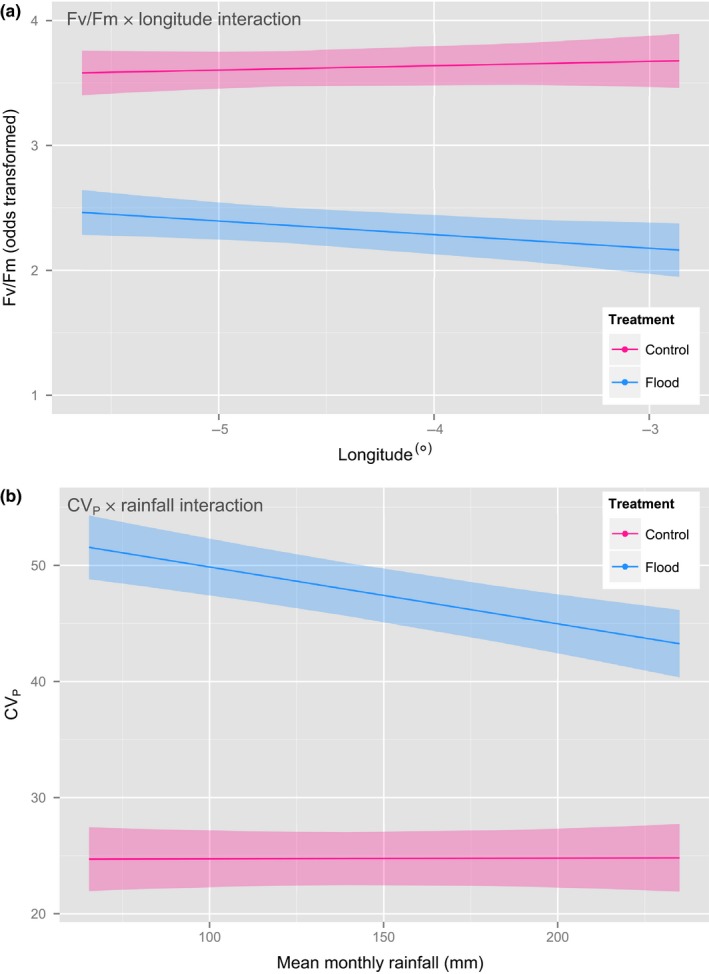
Interaction plots displaying (a) *F*_v_ */F*_m_ (odds‐transformed) × longitude of at site of origin, showing a modest reduction in photochemical capacity under waterlogging with increasing longitude, and (b) CV_P_ × mean monthly rainfall of at site of origin, whereby phenotypic variation under waterlogging increases toward sites with lower rainfall. Data in both plots correspond to current‐year needles [Colour figure can be viewed at http://wileyonlinelibrary.com]

Following the transition to current‐year needles, the treatment effect became more pronounced (ΔAIC_c_ = 163.05); *F*_v_ */F*_m_ values in the flooded group covaried with those in the control, and the difference between groups did not increase with time (Figure 2a). During this period, mean odds‐transformed *F*_v_ */F*_m_ was 4.00 (±0.55) in the control and 1.37 (±0.53) in the flood group (back‐transformed, 0.79 and 0.65, respectively). During this period of measurement, evidence was found for a flood treatment × longitude interaction (ΔAIC_c_ = 3.18); samples from eastern populations experiencing a larger average reduction in *F*_v_ */F*_m_ than those from western populations (Figure 3a).

### 3.3 Effects of flood treatment and sample origin on variation in *F*_v_ */F*_m_ within populations

A small difference in mean CV_P_ for *F*_v_ */F*_m_ between treatment groups emerged over the first 10 weeks (previous‐year needles), similar to the difference in mean *F*_v_ */F*_m_, but in the opposite direction (Figure 2b). During this period, CV_P_ was on average 35.49% (±1.27) and 38.61% (±1.67) for control and flood groups, respectively. Evidence was found for a treatment effect (ΔAIC_c_ = 8.70), but not for covariates longitude or rainfall.

Following commencement of measurement on current‐year needles, the difference in CV_P_ between treatment groups was substantially greater: average CV_P_ being more than twice as high in the flooded treatment (54.78% ± 1.26) than in the control (25.49% ± 0.72) (ΔAIC_c_ = 233.11). Furthermore, there was support for a treatment × rainfall interaction (ΔAIC_c_ = 6.22), whereby CV_P_ was greater on average for sites of origin which receive lower precipitation (Figure 3b).

Mortality throughout the experiment was very low: At the time of completion, two individuals had died, both belonging to the treatment group.

## 4 DISCUSSION

The results of our trial showed that flooding significantly reduced the photosynthetic capacity of PSII in Scots pine saplings from populations from across its range in Scotland. However, plants from populations in the west, where rainfall is higher, showed a lower reduction in the mean value of this proxy of fitness than did plants from populations in the drier east, consistent with local adaptation. The imposition of flooding led to a doubling in the coefficient of variation for the proxy of fitness within all populations, as anticipated if cryptic genetic variation for fitness is expressed under the novel environmental conditions presented by waterlogging. Furthermore, this increase in variation was significantly greater for plants from populations in the east, than those from sites in the west. This is as expected if natural selection, acting upon cryptic genetic variation for flood tolerance, has reduced genetic variance in fitness to flooding within western populations, where exposure to periodic waterlogging is likely to be greatest.

This interpretation of our results is contingent upon the assumption that the dark‐adapted chlorophyll fluorescence parameter *F*_v_ */F*_m_ that we have measured is a reasonable proxy for the long‐term fitness of the plants in the experiment. The argument for using such a measure is that it estimates the capacity of the plant to undertake a critical step in photosynthesis and represents an integrated measure of the ability of the plant to assimilate carbon under the environmental conditions under which it has been placed. Ability to assimilate carbon is likely to be strongly related to ultimate reproductive contribution, and hence fitness.

There is direct evidence from closely related work on the physiological response of Scots pine to waterlogging that changes in *F*_v_ */F*_m_ mirror changes in maximum net assimilation rate of saplings (Repo et al., 2016). Furthermore, a large number of empirical studies indicate that *F*_v_ */F*_m_ responds sensitively and in the direction anticipated to changes in a variety of environmental conditions, such as cold temperature, drought, and flooding that are likely to reduce the fitness of plants (Maxwell & Johnson, 2000; Pearson et al., 2013; Salmela et al., 2011). Moreover, these reductions in *F*_v_ */F*_m_ often differ among populations in ways that are consistent with adaptation to important environmental variation. Thus, while it is clear that *F*_v_ */F*_m_ ratios cannot be directly equated with reproductive fitness, it seems reasonable to assume that they will provide a relative measure of the fitness of a plant under a given set of conditions and that greater variation among plants for this measure will reflect greater variation in fitness.

The second assumption that we have made is that in our experiment the differences in the mean *F*_v_ */F*_m_ value of populations, and in the variance of *F*_v_ */F*_m_ among individuals within populations, have a genetic basis. Common garden studies eliminate as far as possible the environmental component of variation in phenotype, and differences can therefore be assumed to be genetic.

As a species, Scots pine is generally regarded as intolerant of flooding (Glenz, Schlaepfer, Iorgulescu, & Kienast, 2006), and evidence from paleoecology has shown that soil water saturation has been a key factor in limiting its postglacial geographic distribution (Gear & Huntley, 1991). Although it can survive on bogs and does so throughout its range in northern Europe, growth rates are typically very low, and reproduction is not assured and may depend on local drying of peat (Mchaffie, Legg, Worrell, Cowie, & Amphlett, 2002; Mukassabi, Polwart, Coleshaw, & Thomas, 2012; Repo et al., 2016). This is consistent with the results of our flooding exposure, which led to a gradual decline in *F*_v_ */F*_m_ ratios compared with controls in existing needles, and a more marked and constant reduction within the subsequent cohort of needles formed under the flood regime. Comparable results were found by Repo et al. (2016) using 7‐year‐old saplings of Scots pine from Finland, who also recorded many other declines in fitness‐related physiological processes associated with imposition of flooding. Despite the decline in *F*_v_ */F*_m_, mortality recorded throughout the trial was extremely low; this would suggest that the treatment was within plant tolerances, insofar that it was not generally fatal. This is caveated by the fact that the experiment took place under glasshouse conditions in which plants were insulated from competition and other stresses, which they would ordinarily experience upon native sites, and that longer‐term impacts of the treatment on fitness were not evaluated.

In Scotland, where Scots pine exists under the most oceanic climate and under the highest rainfall regime within its natural range, environmental conditions are most likely to favor selection for tolerance of waterlogging. This is particularly true in the west where annual rainfall is greatest (>2,500 mm), and the geology of Torridonian sandstone gives rise to extensive blanket bog formation. In contrast, in the east, where rainfall may be only a third of that in the west and populations are on freely draining gravels derived from Cairngorm granite, flooding of soil is far less prevalent (Mason et al., 2004). Under these conditions, greater waterlogging tolerance is expected to evolve in the western than in the eastern populations.

This prediction is supported by our results, and support was found for a longitudinal effect, with mean *F*_v_ */F*_m_ values under flooding decreasing with distance east. However, the variation in mean *F*_v_ */F*_m_ values with longitude was relatively small. Two factors may account for this. The first is that although there will be selection for greater tolerance in portions of each local population prone to waterlogging, these may make a relatively small genetic contribution to the next generation compared to that fraction of the population growing vigorously on well‐drained soils in the vicinity. Thus, effective selection for greater flood tolerance is only likely in the small subset of western sites where populations are located predominantly on poorly drained soils. The second is that gene flow among populations is high, tending to break down adaptive genetic differentiation among populations. The extent of local adaptation is dependent upon the strength of differential selection relative to the homogenizing effects of gene flow (Kawecki & Ebert, 2004). Scots pine is wind‐pollinated, enabling reproduction across large distances; however, in Scotland, it has been shown that asynchrony in flowering phenology may serve to inhibit gene flow among populations (Whittet, Cavers, Cottrell, Rosique‐Esplugas, & Ennos, 2017). Related work on needle anatomical traits has shown differentiation for stomatal band and resin canal densities, consistent with adaptation to the same longitudinal rainfall gradient that generates adaptive differences in flood tolerance in *P. sylvestris* in Scotland, is also modest (Donnelly et al., 2016).

Apart from reducing mean fitness, the flood treatment led to a doubling in the coefficient of variation in *F*_v_ */F*_m_ *,* our proxy of fitness in the Scots pine populations. The simplest explanation for this is that cryptic genetic variation affecting fitness under flooding has been exposed. The extent of cryptic genetic variation within populations is difficult to predict but is expected to be greatest in outcrossing populations with high effective population size (*N*_e_) (Hermisson & Wagner, 2004). Outcrossing tree populations such as *P. sylvestris*, which are generally large and well connected by extensive gene flow, typically have high *N*_e_ values and therefore are likely to harbor high levels of cryptic genetic variation.

Much concern has been expressed about the ability of such tree populations, with their long generation times, to adapt sufficiently quickly to rapid climate change based on estimates of genetic variance made under current conditions (Hoffmann & Sgrò, 2011). However, the probability of adaptive escape under climate change may be significantly enhanced if genetic variation in fitness under novel environments is increased as a consequence of exposure of cryptic genetic variation (Kopp & Matuszewski, 2014). This suggests that to make more realistic predictions of genetic responses to climate change, we require estimates of quantitative genetic parameters measured in the novel abiotic and biotic environments expected to be encountered under future climates (Ledon‐Rettig et al., 2014).

Apart from the value of *N*_e_, the other main determinant of levels of cryptic genetic variation within populations is the extent to which the population has been exposed in the past to the conditions under which that cryptic variation is expressed (Hermisson & Wagner, 2004). In the present study, we expect populations from the west to have been exposed more regularly than populations in the east to waterlogging and therefore to show less cryptic variation for fitness under flooding. This is supported by our results and is compatible with selection on cryptic genetic variation leading to increase in mean fitness under flooding in the western populations. The fact that cryptic variation can form the basis for genetic changes in the mean values of quantitative traits has already been elegantly demonstrated by Cooper (1954). He showed that artificial selection on cryptic genetic variation for flowering time, which had been exposed by an environmental change in seed vernalization conditions, resulted in rapid genetic divergence between selected lines.

In the absence of climate change, genotypes exist under conditions that are environmentally variable. Here, populations will be under stabilizing selection for phenotypic responses that maximize fitness over the normal range of environments encountered. This leads to the evolution of acclimation responses that may or may not involve phenotypic plasticity, defined merely as a change in the phenotype in response to environmental change (Nicotra et al., 2010). However, even if phenotypic plasticity is shown, this will not generate substantial variation in fitness upon which selection can act within the normal range of conditions, because all genotypes will tend to respond in the same way to any particular environmental change.

Outside the range of normal environments in which the acclimation response operates and has evolved, genotypes encounter conditions to which they have not previously been exposed. Under these conditions, cryptic genetic variation present within the population will lead to individuals showing a variety of phenotypic plasticity responses that are essentially untested by selection (Lande, 2009). The resulting increase in variance in fitness within the population provides additional raw material for adaptation under these novel environmental conditions and determines the rate at which they can evolve (Gibson & Dworkin, 2004; Schlichting, 2008). Thus, the effects of phenotypic plasticity on response to environmental change are not uniform but are critically dependent on context, exposure to the normal or to a novel range of environments (Lande, 2009).

These arguments indicate that rather than contrasting the roles of phenotypic plasticity and natural selection in the response of populations to rapid climate change, we should be more concerned with comparing the effects of acclimation and cryptic genetic variation (Nicotra et al., 2010; Kopp & Matuszewski, 2013). Furthermore, if we are to understand the long‐term evolutionary responses to climate change, it would appear that estimates of genetic parameters made within the normal range of environments encountered by present‐day populations may not be sufficient. Instead, additional experimental quantitative genetic research programs are required to uncover the cryptic genetic variation exposed in the novel environments anticipated under rapid climate change (Ledon‐Rettig et al., 2014).

## CONFLICT OF INTEREST

None declared.

## AUTHORS' CONTRIBUTIONS

KD, JC, RAE, and SC conceived this study. KD performed laboratory work and analysis. KD wrote the manuscript, with input and revisions from JC, RAE, and SC.

## DATA ARCHIVING STATEMENT

Chlorophyll fluorescence data will be deposited in the Environmental Information Data Centre (EIDC) upon acceptance.
