# Supplementary material for: Cryptic genetic variation and adaptation to waterlogging in Caledonian Scots pine, Pinus sylvestris L
Source: Ecol Evol. 2018 Aug 2;8(17):8665–75. doi: 10.1002/ece3.4389 (PMC6157661; doi:10.1002/ece3.4389)
Supplement: Supplementary file 1 [file ECE3-8-8665-s001.docx]

**Table S1**: Model subset tables for differing combinations of fixed effects. For any given model the terms present are represent by either ‘+’ (for factors), or the value of the coefficient (for covariates); the absence of a parameter is denoted ‘-‘. Note that ‘DAY’ was standardised (divided by two standard deviations) prior to analyses. Longitude and Rainfall terms were not permitted to occur within the same model. Tables 1 (a-b) describe fixed effects for the original data; (c-d) for CV_P_ data (estimated at the population level).

1a) Chlorophyll Fluorescence: Odd Transformed Fv/Fm for previous-year needles

| Intercept | TRE | LONG | RAIN | DAY | TRE:LONG | TRE:RAIN | TRE:DAY | LONG:DAY | RAIN:DAY | df | logLik | AICc | ΔAICc | *w* |
| --- | --- | --- | --- | --- | --- | --- | --- | --- | --- | --- | --- | --- | --- | --- |
| 3.470 | + | - | - | -1.32E-01 | - | - | + | - | - | 10 | -3887.96 | 7796.0 | 0.00 | 0.391 |
| 3.442 | + | -6.22E-03 | - | -1.32E-01 | - | - | + | - | - | 11 | -3887.95 | 7798.0 | 2.00 | 0.144 |
| 3.459 | + | - | 7.58E-05 | -1.32E-01 | - | - | + | - | - | 11 | -3887.96 | 7798.0 | 2.00 | 0.143 |
| 3.59 | + | 2.68E-02 | - | -1.32E-01 | + | - | + | - | - | 12 | -3887.48 | 7799.1 | 3.07 | 0.084 |
| 3.335 | + | -2.98E-02 | - | 2.81E-03 | - | - | + | 2.98E-02 | - | 12 | -3887.72 | 7799.6 | 3.55 | 0.066 |
| 3.421 | + | - | 3.37E-04 | -8.44E-02 | - | - | + | - | -3.31E-04 | 12 | -3887.83 | 7799.8 | 3.77 | 0.059 |
| 3.457 | + | - | 9.05E-05 | -1.32E-01 | - | + | + | - | - | 12 | -3887.96 | 7800.0 | 4.02 | 0.052 |
| 3.484 | + | 3.24E-03 | - | 2.75E-03 | + | - | + | 2.98E-02 | - | 13 | -3887.25 | 7800.6 | 4.63 | 0.039 |
| 3.419 | + | - | 3.52E-04 | -8.44E-02 | - | + | + | - | -3.31E-04 | 13 | -3887.83 | 7801.8 | 5.79 | 0.022 |
| 3.664 | + | - | - | -3.78E-01 | - | - | - | - | - | 9 | -3906.34 | 7830.8 | 34.74 | 0.000 |
| 3.635 | + | -6.40E-03 | - | -3.78E-01 | - | - | - | - | - | 10 | -3906.33 | 7832.8 | 36.74 | 0.000 |
| 3.653 | + | - | 7.82E-05 | -3.78E-01 | - | - | - | - | - | 10 | -3906.34 | 7832.8 | 36.75 | 0.000 |
| 3.364 | + | - | - | - | - | - | - | - | - | 8 | -3908.81 | 7833.7 | 37.67 | 0.000 |
| 3.783 | + | 2.64E-02 | - | -3.78E-01 | + | - | - | - | - | 11 | -3905.87 | 7833.8 | 37.83 | 0.000 |
| 3.523 | + | -3.12E-02 | - | -2.36E-01 | - | - | - | 3.14E-02 | - | 11 | -3906.08 | 7834.3 | 38.24 | 0.000 |
| 3.612 | + | - | 3.58E-04 | -3.27E-01 | - | - | - | - | -3.55E-04 | 11 | -3906.19 | 7834.5 | 38.48 | 0.000 |
| 3.65 | + | - | 9.67E-05 | -3.78E-01 | - | + | - | - | - | 11 | -3906.34 | 7834.8 | 38.76 | 0.000 |
| 3.671 | + | 1.55E-03 | - | -2.36E-01 | + | - | - | 3.14E-02 | - | 12 | -3905.61 | 7835.3 | 39.34 | 0.000 |
| 3.335 | + | -6.39E-03 | - | - | - | - | - | - | - | 9 | -3908.80 | 7835.7 | 39.67 | 0.000 |
| 3.353 | + | - | 7.78E-05 | - | - | - | - | - | - | 9 | -3908.81 | 7835.7 | 39.67 | 0.000 |
| 3.61 | + | - | 3.78E-04 | -3.27E-01 | - | + | - | - | -3.55E-04 | 12 | -3906.19 | 7836.5 | 40.49 | 0.000 |
| 3.483 | + | 2.64E-02 | - | - | + | - | - | - | - | 10 | -3908.34 | 7836.8 | 40.75 | 0.000 |
| 3.35 | + | - | 9.61E-05 | - | - | + | - | - | - | 10 | -3908.81 | 7837.7 | 41.69 | 0.000 |
| 3.466 | - | - | - | -3.77E-01 | - | - | - | - | - | 8 | -3916.44 | 7848.9 | 52.92 | 0.000 |
| 3.441 | - | -5.65E-03 | - | -3.77E-01 | - | - | - | - | - | 9 | -3916.43 | 7850.9 | 54.92 | 0.000 |
| 3.457 | - | - | 6.69E-05 | -3.77E-01 | - | - | - | - | - | 9 | -3916.43 | 7850.9 | 54.92 | 0.000 |
| 3.166 | - | - | - | - | - | - | - | - | - | 7 | -3918.81 | 7851.7 | 55.66 | 0.000 |
| 3.328 | - | -3.06E-02 | - | -2.35E-01 | - | - | - | 3.16E-02 | - | 10 | -3916.17 | 7852.4 | 56.42 | 0.000 |
| 3.416 | - | - | 3.49E-04 | -3.26E-01 | - | - | - | - | -3.57E-04 | 10 | -3916.29 | 7852.7 | 56.65 | 0.000 |
| 3.141 | - | -5.64E-03 | - | - | - | - | - | - | - | 8 | -3918.81 | 7853.7 | 57.66 | 0.000 |
| 3.157 | - | - | 6.65E-05 | - | - | - | - | - | - | 8 | -3918.81 | 7853.7 | 57.66 | 0.000 |

1b) Chlorophyll Fluorescence: Odd Transformed Fv/Fm for current-year needles

| Intercept | TRE | LONG | RAIN | DAY | TRE:LONG | TRE:RAIN | TRE:DAY | LONG:DAY | RAIN:DAY | df | logLik | AICc | ΔAICc | *w* |
| --- | --- | --- | --- | --- | --- | --- | --- | --- | --- | --- | --- | --- | --- | --- |
| 3.721 | + | -2.60E-02 | - | 4.19E-02 | + | - | + | 4.69E-02 | - | 13 | -5547.00 | 11120.1 | 0.00 | 0.326 |
| 3.995 | + | 3.47E-02 | - | -1.70E-01 | + | - | + | - | - | 12 | -5548.63 | 11121.3 | 1.26 | 0.174 |
| 3.707 | + | - | 9.18E-04 | -1.70E-01 | - | + | + | - | - | 12 | -5549.05 | 11122.2 | 2.10 | 0.114 |
| 3.838 | + | - | - | -1.70E-01 | - | - | + | - | - | 10 | -5551.33 | 11122.7 | 2.63 | 0.088 |
| 3.595 | + | - | 0.001696 | -1.70E-01 | - | - | + | - | - | 11 | -5550.40 | 11122.9 | 2.77 | 0.082 |
| 3.636 | + | - | 1.41E-03 | -1.15E-01 | - | + | + | - | -3.79E-04 | 13 | -5548.59 | 11123.3 | 3.18 | 0.067 |
| 3.398 | + | -9.76E-02 | - | 4.22E-02 | - | - | + | 4.70E-02 | - | 12 | -5549.59 | 11123.3 | 3.18 | 0.066 |
| 3.525 | + | - | 0.002188 | -1.15E-01 | - | - | + | - | -3.81E-04 | 12 | -5549.93 | 11123.9 | 3.84 | 0.048 |
| 3.672 | + | -3.69E-02 | - | -1.70E-01 | - | - | + | - | - | 11 | -5551.24 | 11124.5 | 4.45 | 0.035 |
| 3.776 | + | 3.48E-02 | - | - | + | - | - | - | - | 10 | -5613.15 | 11246.4 | 126.28 | 0.000 |
| 3.369 | + | -2.45E-02 | - | 3.14E-01 | + | - | - | 4.59E-02 | - | 12 | -5611.45 | 11247.0 | 126.90 | 0.000 |
| 3.487 | + | - | 9.17E-04 | - | - | + | - | - | - | 10 | -5613.57 | 11247.2 | 127.12 | 0.000 |
| 3.619 | + | - | - | - | - | - | - | - | - | 8 | -5615.88 | 11247.8 | 127.72 | 0.000 |
| 3.375 | + | - | 0.001703 | - | - | - | - | - | - | 9 | -5614.94 | 11247.9 | 127.85 | 0.000 |
| 3.637 | + | 3.48E-02 | - | 1.07E-01 | + | - | - | - | - | 11 | -5612.96 | 11248.0 | 127.91 | 0.000 |
| 3.349 | + | - | 9.17E-04 | 1.07E-01 | - | + | - | - | - | 11 | -5613.38 | 11248.8 | 128.75 | 0.000 |
| 3.48 | + | - | - | 1.07E-01 | - | - | - | - | - | 9 | -5615.69 | 11249.4 | 129.35 | 0.000 |
| 3.236 | + | - | 0.001703 | 1.07E-01 | - | - | - | - | - | 10 | -5614.75 | 11249.6 | 129.48 | 0.000 |
| 3.451 | + | -3.72E-02 | - | - | - | - | - | - | - | 9 | -5615.79 | 11249.6 | 129.54 | 0.000 |
| 3.282 | + | - | 1.38E-03 | 1.59E-01 | - | + | - | - | -3.61E-04 | 12 | -5612.98 | 11250.0 | 129.95 | 0.000 |
| 3.044 | + | -9.66E-02 | - | 3.15E-01 | - | - | - | 4.60E-02 | - | 11 | -5614.08 | 11250.2 | 130.15 | 0.000 |
| 3.169 | + | - | 0.002171 | 1.59E-01 | - | - | - | - | -3.62E-04 | 11 | -5614.34 | 11250.7 | 130.67 | 0.000 |
| 3.312 | + | -3.72E-02 | - | 1.07E-01 | - | - | - | - | - | 10 | -5615.60 | 11251.2 | 131.17 | 0.000 |
| 2.98 | - | - | - | - | - | - | - | - | - | 7 | -5633.32 | 11280.7 | 160.59 | 0.000 |
| 2.735 | - | - | 0.001706 | - | - | - | - | - | - | 8 | -5632.41 | 11280.9 | 160.78 | 0.000 |
| 2.841 | - | - | - | 1.07E-01 | - | - | - | - | - | 8 | -5633.14 | 11282.3 | 162.23 | 0.000 |
| 2.811 | - | -3.74E-02 | - | - | - | - | - | - | - | 8 | -5633.23 | 11282.5 | 162.41 | 0.000 |
| 2.596 | - | - | 0.001706 | 1.07E-01 | - | - | - | - | - | 9 | -5632.23 | 11282.5 | 162.42 | 0.000 |
| 2.405 | - | -9.67E-02 | - | 3.14E-01 | - | - | - | 4.59E-02 | - | 10 | -5631.54 | 11283.1 | 163.05 | 0.000 |
| 2.53 | - | - | 0.002172 | 1.59E-01 | - | - | - | - | -3.61E-04 | 10 | -5631.82 | 11283.7 | 163.61 | 0.000 |
| 2.672 | - | -3.74E-02 | - | 1.07E-01 | - | - | - | - | - | 9 | -5633.05 | 11284.1 | 164.06 | 0.000 |

1c) Chlorophyll Fluorescence: CV_P_ for previous-year needles

| Intercept | TRE | LONG | RAIN | DAY | TRE:LONG | TRE:RAIN | TRE:DAY | LONG:DAY | RAIN:DAY | df | logLik | AICc | ΔAICc | *w* |
| --- | --- | --- | --- | --- | --- | --- | --- | --- | --- | --- | --- | --- | --- | --- |
| 35.49 | + | - | - | 6.35E+00 | - | - | + | - | - | 7 | -385.01 | 785.1 | 0.00 | 0.346 |
| 37.35 | + | - | -1.30E-02 | 6.35E+00 | - | + | + | - | - | 9 | -383.63 | 787.1 | 1.97 | 0.129 |
| 34.41 | + | - | 7.53E-03 | 6.35E+00 | - | - | + | - | - | 8 | -384.95 | 787.4 | 2.22 | 0.114 |
| 35.78 | + | 6.35E-02 | - | 6.35E+00 | - | - | + | - | - | 8 | -385.01 | 787.5 | 2.33 | 0.108 |
| 37.35 | + | - | -1.30E-02 | 1.70E+00 | - | + | + | - | 3.24E-02 | 10 | -382.80 | 787.9 | 2.73 | 0.088 |
| 34.41 | + | - | 7.53E-03 | 1.70E+00 | - | - | + | - | 3.24E-02 | 9 | -384.14 | 788.1 | 2.98 | 0.078 |
| 35.78 | + | 6.35E-02 | - | -1.41E+00 | - | - | + | -1.72E+00 | - | 9 | -384.49 | 788.8 | 3.67 | 0.055 |
| 36.11 | + | 1.37E-01 | - | 6.35E+00 | + | - | + | - | - | 9 | -385.00 | 789.8 | 4.71 | 0.033 |
| 36.11 | + | 1.37E-01 | - | -1.41E+00 | + | - | + | -1.72E+00 | - | 10 | -384.48 | 791.2 | 6.10 | 0.016 |
| 35.49 | + | - | - | 1.14E+01 | - | - | - | - | - | 6 | -389.87 | 792.6 | 7.44 | 0.008 |
| 37.05 | - | - | - | 1.14E+01 | - | - | - | - | - | 5 | -391.62 | 793.8 | 8.70 | 0.004 |
| 37.35 | + | - | -1.30E-02 | 1.14E+01 | - | + | - | - | - | 8 | -388.62 | 794.7 | 9.56 | 0.003 |
| 34.41 | + | - | 7.53E-03 | 1.14E+01 | - | - | - | - | - | 7 | -389.82 | 794.7 | 9.61 | 0.003 |
| 35.78 | + | 6.35E-02 | - | 1.14E+01 | - | - | - | - | - | 7 | -389.87 | 794.9 | 9.73 | 0.003 |
| 37.35 | + | - | -1.30E-02 | 6.75E+00 | - | + | - | - | 3.24E-02 | 9 | -387.87 | 795.6 | 10.43 | 0.002 |
| 34.41 | + | - | 7.53E-03 | 6.75E+00 | - | - | - | - | 3.24E-02 | 8 | -389.08 | 795.6 | 10.47 | 0.002 |
| 35.97 | - | - | 7.53E-03 | 1.14E+01 | - | - | - | - | - | 6 | -391.57 | 796.0 | 10.82 | 0.002 |
| 37.34 | - | 6.35E-02 | - | 1.14E+01 | - | - | - | - | - | 6 | -391.62 | 796.1 | 10.94 | 0.001 |
| 35.78 | + | 6.35E-02 | - | 3.63E+00 | - | - | - | -1.72E+00 | - | 8 | -389.40 | 796.3 | 11.12 | 0.001 |
| 35.97 | - | - | 7.53E-03 | 6.75E+00 | - | - | - | - | 3.24E-02 | 7 | -390.85 | 796.8 | 11.69 | 0.001 |
| 36.11 | + | 1.37E-01 | - | 1.14E+01 | + | - | - | - | - | 8 | -389.87 | 797.2 | 12.05 | 0.001 |
| 37.34 | - | 6.35E-02 | - | 3.63E+00 | - | - | - | -1.72E+00 | - | 7 | -391.17 | 797.5 | 12.31 | 0.001 |
| 36.11 | + | 1.37E-01 | - | 3.63E+00 | + | - | - | -1.72E+00 | - | 9 | -389.40 | 798.6 | 13.49 | 0.000 |
| 35.49 | + | - | - | - | - | - | - | - | - | 5 | -396.56 | 803.7 | 18.57 | 0.000 |
| 37.05 | - | - | - | - | - | - | - | - | - | 4 | -398.30 | 805.0 | 19.85 | 0.000 |
| 37.35 | + | - | -1.30E-02 | - | - | + | - | - | - | 7 | -395.32 | 805.8 | 20.62 | 0.000 |
| 34.41 | + | - | 7.53E-03 | - | - | - | - | - | - | 6 | -396.51 | 805.8 | 20.71 | 0.000 |
| 35.78 | + | 6.35E-02 | - | - | - | - | - | - | - | 6 | -396.56 | 806.0 | 20.81 | 0.000 |
| 35.97 | - | - | 7.53E-03 | - | - | - | - | - | - | 5 | -398.25 | 807.1 | 21.95 | 0.000 |
| 37.34 | - | 6.35E-02 | - | - | - | - | - | - | - | 5 | -398.30 | 807.2 | 22.05 | 0.000 |
| 36.11 | + | 1.37E-01 | - | - | + | - | - | - | - | 7 | -396.56 | 808.2 | 23.09 | 0.000 |

1d) Chlorophyll Fluorescence: CV_P_ for current-year needles

| Intercept | TRE | LONG | RAIN | DAY | TRE:LONG | TRE:RAIN | TRE:DAY | LONG:DAY | RAIN:DAY | df | logLik | AICc | ΔAICc | *w* |
| --- | --- | --- | --- | --- | --- | --- | --- | --- | --- | --- | --- | --- | --- | --- |
| 24.68 | + | - | 6.68E-04 | 1.76E+00 | - | + | + | - | - | 9 | -623.02 | 1265.1 | 0.00 | 0.493 |
| 24.68 | + | - | 6.68E-04 | 1.56E-01 | - | + | + | - | 1.12E-02 | 10 | -622.80 | 1266.9 | 1.80 | 0.200 |
| 21.61 | + | -7.01E-01 | - | 1.76E+00 | + | - | + | - | - | 9 | -624.08 | 1267.2 | 2.13 | 0.170 |
| 21.61 | + | -7.01E-01 | - | -1.23E+00 | + | - | + | -6.65E-01 | - | 10 | -623.91 | 1269.1 | 4.02 | 0.066 |
| 24.78 | + | - | - | 1.76E+00 | - | - | + | - | - | 7 | -628.22 | 1271.1 | 6.00 | 0.025 |
| 28.23 | + | - | -2.41E-02 | 1.76E+00 | - | - | + | - | - | 8 | -627.24 | 1271.3 | 6.22 | 0.022 |
| 28.7 | + | 8.71E-01 | - | 1.76E+00 | - | - | + | - | - | 8 | -627.95 | 1272.7 | 7.65 | 0.011 |
| 28.23 | + | - | -2.41E-02 | 1.56E-01 | - | - | + | - | 1.12E-02 | 9 | -627.03 | 1273.1 | 8.02 | 0.009 |
| 28.7 | + | 8.71E-01 | - | -1.23E+00 | - | - | + | -6.65E-01 | - | 9 | -627.79 | 1274.6 | 9.53 | 0.004 |
| 24.68 | + | - | 6.68E-04 | -5.93E+00 | - | + | - | - | - | 8 | -646.47 | 1309.8 | 44.68 | 0.000 |
| 24.68 | + | - | 6.68E-04 | - | - | + | - | - | - | 7 | -647.65 | 1310.0 | 44.86 | 0.000 |
| 24.68 | + | - | 6.68E-04 | -7.54E+00 | - | + | - | - | 1.12E-02 | 9 | -646.30 | 1311.7 | 46.56 | 0.000 |
| 21.61 | + | -7.01E-01 | - | -5.93E+00 | + | - | - | - | - | 8 | -647.44 | 1311.7 | 46.63 | 0.000 |
| 21.61 | + | -7.01E-01 | - | - | + | - | - | - | - | 7 | -648.63 | 1311.9 | 46.81 | 0.000 |
| 21.61 | + | -7.01E-01 | - | -8.93E+00 | + | - | - | -6.65E-01 | - | 9 | -647.31 | 1313.7 | 48.58 | 0.000 |
| 24.78 | + | - | - | -5.93E+00 | - | - | - | - | - | 6 | -650.63 | 1313.7 | 48.65 | 0.000 |
| 28.23 | + | - | -2.41E-02 | -5.93E+00 | - | - | - | - | - | 7 | -649.64 | 1313.9 | 48.84 | 0.000 |
| 24.78 | + | - | - | - | - | - | - | - | - | 5 | -651.81 | 1314.0 | 48.87 | 0.000 |
| 28.23 | + | - | -2.41E-02 | - | - | - | - | - | - | 6 | -650.83 | 1314.2 | 49.06 | 0.000 |
| 28.7 | + | 8.71E-01 | - | -5.93E+00 | - | - | - | - | - | 7 | -650.36 | 1315.4 | 50.27 | 0.000 |
| 28.7 | + | 8.71E-01 | - | - | - | - | - | - | - | 6 | -651.54 | 1315.6 | 50.48 | 0.000 |
| 28.23 | + | - | -2.41E-02 | -7.54E+00 | - | - | - | - | 1.12E-02 | 8 | -649.49 | 1315.8 | 50.72 | 0.000 |
| 28.7 | + | 8.71E-01 | - | -8.93E+00 | - | - | - | -6.65E-01 | - | 8 | -650.23 | 1317.3 | 52.21 | 0.000 |
| 36.26 | - | - | - | -5.93E+00 | - | - | - | - | - | 5 | -743.79 | 1497.9 | 232.83 | 0.000 |
| 39.71 | - | - | -2.41E-02 | -5.93E+00 | - | - | - | - | - | 6 | -742.86 | 1498.2 | 233.11 | 0.000 |
| 36.26 | - | - | - | - | - | - | - | - | - | 4 | -744.99 | 1498.2 | 233.12 | 0.000 |
| 39.71 | - | - | -2.41E-02 | - | - | - | - | - | - | 5 | -744.07 | 1498.5 | 233.38 | 0.000 |
| 40.18 | - | 8.71E-01 | - | -5.93E+00 | - | - | - | - | - | 6 | -743.52 | 1499.5 | 234.42 | 0.000 |
| 40.18 | - | 8.71E-01 | - | - | - | - | - | - | - | 5 | -744.72 | 1499.8 | 234.70 | 0.000 |
| 39.71 | - | - | -2.41E-02 | -7.54E+00 | - | - | - | - | 1.12E-02 | 7 | -742.81 | 1500.3 | 235.17 | 0.000 |
| 40.18 | - | 8.71E-01 | - | -8.93E+00 | - | - | - | -6.65E-01 | - | 7 | -743.48 | 1501.6 | 236.51 | 0.000 |

**Table S2**: Model output containing parameter estimates for each of the top AICc models described above. Note that ‘DAY’ was standardised (divided by two standard deviations) prior to analyses. Tables 2(a-b) describe output for odds-transformed Fv/Fm data, (c-d) for CV_P_ which was estimates at population level. All models were fitted using REML.

2a) Odds-transformed Fv/Fm for previous-year needles

Linear mixed model fit by REML ['lmerMod']

Formula: Odd_FvFm ~ TREATMENT * DAY + (1 | DAY) + (1 | POP/FAM/IND) + (1 | PLOT)

REML criterion at convergence: 7787.884

Random effects:

Groups Name Std.Dev.

IND:(FAM:POP) (Intercept) 0.51827

FAM:POP (Intercept) 0.12588

PLOT (Intercept) 0.00000

POP (Intercept) 0.05038

DAY (Intercept) 0.19436

Residual 1.02252

Number of obs: 2551, groups: IND:(FAM:POP), 430; FAM:POP, 36; PLOT, 12; POP, 9; DAY, 6

Fixed Effects:

(Intercept) TREATMENTF DAY TREATMENTF:DAY

3.46960 -0.00603 -0.13183 -0.49376

2b) Odds-transformed Fv/Fm for current-year needles

Linear mixed model fit by REML ['lmerMod']

Formula: Odd_FvFm ~ TREATMENT * LONG + TREATMENT * DAY + LONG * DAY + (1 | DAY) + (1 | POP/FAM/IND) + (1 | PLOT)

REML criterion at convergence: 11117.28

Random effects:

Groups Name Std.Dev.

IND:(FAM:POP) (Intercept) 0.5540

FAM:POP (Intercept) 0.1877

PLOT (Intercept) 0.1164

DAY (Intercept) 0.2966

POP (Intercept) 0.2302

Residual 0.7955

Number of obs: 4307, groups: IND:(FAM:POP), 432; FAM:POP, 36; PLOT, 12; DAY, 10; POP, 9

Fixed Effects:

(Intercept) TREATMENTF LONG DAY TREATMENTF:LONG TREATMENTF:DAY LONG:DAY

3.72102 -2.64183 -0.02603 0.04185 -0.14333 0.55527 0.04693

2c) CV_P_ for previous-year needles

Linear mixed model fit by REML ['lmerMod']

Formula: CVP ~ TREATMENT * DAY + (1 | DAY) + (1 | POP)

REML criterion at convergence: 757.6931

Random effects:

Groups Name Std.Dev.

POP (Intercept) 3.670

DAY (Intercept) 1.015

Residual 8.240

Number of obs: 108, groups: POP, 9; DAY, 6

Fixed Effects:

(Intercept) TREATMENTF DAY TREATMENTF:DAY

35.494 3.113 6.346 10.089

2d) CV_P_ for current-year needles

Linear mixed model fit by REML ['lmerMod']

Formula: CVP ~ TREATMENT * RAIN + TREATMENT * DAY + (1 | DAY_R) + (1 | POP)

REML criterion at convergence: 1245.604

Random effects:

Groups Name Std.Dev.

DAY (Intercept) 6.109

POP (Intercept) 2.855

Residual 7.047

Number of obs: 180, groups: DAY, 10; POP, 9

Fixed Effects:

(Intercept) TREATMENTF RAIN DAY TREATMENTF:RAIN TREATMENTF:DAY

2.468e+01 3.007e+01 6.676e-04 1.764e+00 -4.956e-02 -1.540e+01
